# Relationship between psychological distress, health behaviours and future reports of hypertension among adults in East Zimbabwe: a cohort study

**DOI:** 10.1136/openhrt-2023-002346

**Published:** 2023-06-29

**Authors:** Shehla Shamsuddin, Katherine Davis, Louisa Moorhouse, Phyllis Mandizvidza, Rufurwokuda Maswera, Tawanda Dadirai, Constance Nyamukapa, Simon Gregson, Sungano Chigogora

**Affiliations:** 1 Department of Health and Social Care, UK Government, London, UK; 2 MRC Centre for Global Infectious Disease Analysis, Imperial College London, London, UK; 3 Manicaland Centre for Public Health Research, Biomedical Research and Training Institute, Harare, Zimbabwe

**Keywords:** hypertension, epidemiology, public health, global health, biostatistics

## Abstract

**Introduction:**

Extensive cross-sectional evidence has demonstrated an association between psychological distress (PD) and hypertension. However, evidence on the temporal relationship is limited, especially in low-income and middle-income countries. The role of health risk behaviours including smoking and alcohol consumption in this relationship is also largely unknown. The aim of this study was to investigate the association between PD and later development of hypertension, and how this association may have been influenced by health risk behaviours, among adults in east Zimbabwe.

**Methods:**

The analysis included 742 adults (aged 15–54 years) recruited by the Manicaland general population cohort study, who did not have hypertension at baseline in 2012–2013, and who were followed until 2018-2019. In 2012–2013, PD was measured using the Shona Symptom Questionnaire, a screening tool validated for use in Shona-speaking countries including Zimbabwe (cut-off point: 7). Smoking, alcohol consumption and use of drugs (health risk behaviours) were also self-reported. In 2018-2019, participants reported if they had diagnosed with hypertension by a doctor or nurse. Logistic regression was used to assess the association between PD and hypertension.

**Results:**

In 2012, 10.4% of the participants had PD. The odds of new reports of hypertension were 2.04 times greater (95% CI 1.16 to 3.59) among those with PD at baseline, after adjusting for sociodemographic and health risk behaviour variables. Female gender (adjusted odds ratio, AOR 6.89, 95% CI 2.71 to 17.53), older age (AOR 2.67, 95% CI 1.63 to 4.42), and greater wealth (AOR 2.10, 95% CI 1.04 to 4.24 more wealthy, 2.88, 95% CI 1.24 to 6.67 most wealthy) were significant risk factors for hypertension. The AOR for the relationship between PD and hypertension did not differ substantially between models with and without health risk behaviours.

**Conclusion:**

PD was associated with an increased risk of later reports of hypertension in the Manicaland cohort. Integrating mental health and hypertension services within primary healthcare may reduce the dual burden of these non-communicable diseases.

**WHAT IS ALREADY KNOWN ON THIS TOPIC**

- Evidence from a range of settings indicates that there is a cross-sectional relationship between psychological distress (PD) and hypertension.
- Similar evidence also suggests that health risk behaviours like smoking, drugs and alcohol may play a role in this relationship.
- However, there are limited data on the possible temporal relationship between PD and later development of hypertension in sub-Saharan African countries such as Zimbabwe.

**WHAT THIS STUDY ADDS**

- We investigated the temporal association between PD and later development of hypertension in Manicaland, Zimbabwe.
- Individuals with PD at baseline had twice the odds of reporting a hypertension diagnosis compared with individuals without PD, after an average of 6 years of follow-up (adjusted OR 2.04, 95% CI 1.16 to 3.59).
- There was no strong evidence that either smoking status, drug use or alcohol consumption contributed to this relationship.

**HOW THIS STUDY MIGHT AFFECT RESEARCH, PRACTICE OR POLICY**

- Our findings suggest that mental health interventions could be beneficial for reducing hypertension in Zimbabwe.

## Introduction

Hypertension, a major modifiable risk factor for cardiovascular disease, continues to be the leading modifiable cause of death globally, responsible for approximately 10.4 million deaths per year.^1^ It is characterised by elevated blood pressure, which can be defined as systolic blood pressure greater than or equal to 140 mm Hg and/or diastolic blood pressure greater than or equal to 90 mm Hg.^2^ While hypertension is prevalent in both high-income countries and low- and middle-income countries (LMICs), it disproportionately affects populations living in LMICs, including those in sub-Saharan Africa, where access to diagnosis and treatment is more limited.^3 4^

Given the severe health consequences of hypertension, it is important to understand risk factors for the disease. One factor that could influence whether someone develops hypertension is psychological distress (PD).^5–8^ PD is a state of emotional suffering characterised by difficulty coping with daily life stressors; it is manifested in symptoms of depression and anxiety which often occur together.^9^ The coexistence of hypertension and PD is frequently discussed in the public health literature.^5–8 10^ However, there is limited research, especially in sub-Saharan Africa, on the temporal association between PD and future reports of hypertension. One 5-year longitudinal study of normotensive Black South Africans reported twice the risk of subsequent development of hypertension among people with PD.^11^ Yet these results have not been confirmed in other settings in sub-Saharan Africa.

There are several sociodemographic and behavioural factors that may contribute to the prevalence of both PD and hypertension at the individual level (genetic factors, age, gender), household level (income, expenditure, wealth) and societal level (neighbourhood, stigma, poverty and ethnicity).^12 13^ Behavioural risk factors, including smoking, physical inactivity, high salt consumption, alcohol consumption and use of drugs, may be associated with PD and hypertension, and may also be on the causal pathway from PD to development of hypertension.^14^ Notably, a predictor of cardiovascular disease among the black South African population described above was alcohol consumption.^11^ Understanding the role of sociodemographic and behavioural risk factors will support identification of strategies and interventions to reduce the burden of hypertension.

Most of the evidence for links between PD, sociodemographic and behavioural risk factors, and hypertension in sub-Saharan Africa is, however, from South Africa, due to a lack of availability of data elsewhere. An exception to this is Manicaland Province, Zimbabwe, where extensive relevant data has been collected longitudinally from adult men and women through the Manicaland HIV/Sexually Transmitted Disease Prevention Project (Manicaland study). Therefore, analysis of Manicaland study data is the focus of this study. In the Zimbabwean context, pre-existing research can give an insight into the burden of PD and hypertension. A small study of adults attending a primary healthcare facility in Harare, performed in 2013, estimated the prevalence of anxiety and depression to be over 20%.^15 16^ In Manicaland province, the prevalence of PD, assessed between 2009 and 2011, was 4.5% (95% CI 3.9% to 5.1%) among men and 12.9% (95% CI 12.2% to 13.6%) among women.^17^ Moreover, a systematic review of hypertension in Zimbabwe (1980–2013) showed that the prevalence of hypertension may be as high as 30%, with a higher prevalence of hypertension among women, overweight or obese people, people from low income households and people with high alcohol consumption.^18^ The substantial burdens of both hypertension and PD demonstrate the urgent need to better understand links between the two conditions in Zimbabwe.

In this study, we investigated the association between PD and later development of hypertension and assessed the role of health risk behaviours in the relationship between PD and hypertension.

## Methods

### Sampling design and population

Data for this analysis were taken from the ongoing Manicaland study, a general population cohort survey initiated in Manicaland province (eastern Zimbabwe) in 1998. Details on the rationale, design, sampling and data collection methods are described in detail elsewhere.^19–21^ Of the seven rounds of data collection conducted in the Manicaland study, we selected data from round 6 as the baseline round (2012–2013), and round 7 as the follow-up round (2018–2019), because hypertension was assessed in detail for the first time in round 7.^22^

**Figure 1:**
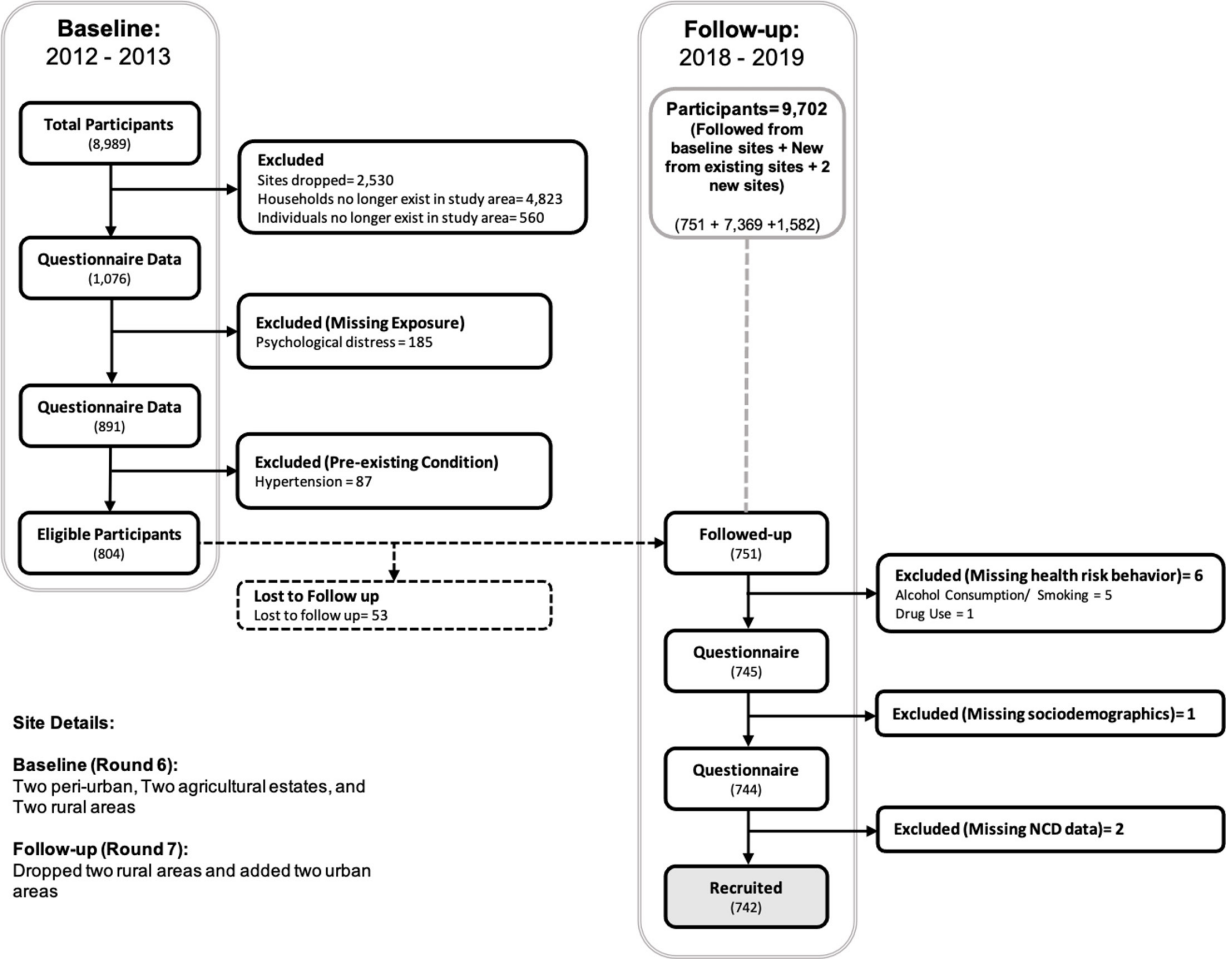
Flow diagram for the selection of the study participants. This figure depicts the criteria that were applied in selecting the study participants and the numbers that were affected by these criteria. NCD, non-communicable disease.

A total of 891 adults, aged 15–54 years, who were surveyed in the 2012–2013 Manicaland survey were still living in eligible study sites in 2018–2019. Between baseline and follow-up two rural sites were dropped from the survey, but the remaining sites continued to represent periurban, agricultural estate and rural areas. Participants were not eligible for this study if they had missing exposure data (n=185), reported pre-existing hypertension at baseline (n=87) or were lost to follow-up (n=53). Figure 1 illustrates how our study sample was selected from the Manicaland survey. Of 751 participants followed up in 2018–2019, 9 had missing covariate or outcome data; this left a total analytical sample 742 participants.

### Data collection

Participants completed a questionnaire (available on the Manicaland study website: http://www.manicalandhivproject.org/questionnaires) with the help of research assistants who were social science graduates.^21^ In the baseline survey, the questionnaire was administered through EpiCollect software on smartphones. In the follow-up survey, the questionnaire was competed on tablets or smart phones using Open Data Kit Collect V.1.0 software.^23^

Participation was voluntary, and all participants provided written informed consent; moreover, they were free to withdraw from the study any time without owing any explanation. The anonymity of the data was maintained by assigning study site numbers, household numbers and a unique reference number to participants, instead of using identifying data, to ensure participants’ confidentiality.

### Measures and procedures

PD at baseline was assessed using the Shona Symptom Questionnaire (SSQ), a screening tool validated for use in Shona speaking countries including Zimbabwe, which is composed of 14 ‘yes or no’ questions.^24^ The SSQ has high internal consistency and it has been widely used, including among patients accessing primary care or mental health support, adolescents and young people, and pregnant women.^15 24–28^ Most SSQ questions focus on symptoms that are common in tools for measuring PD and depression, such as sleep disturbance and suicidal thoughts, however, others focus on local idioms for PD, such as ‘thinking deeply or thinking about many things’. Participants are asked if they have experienced each symptom in the last week. A cut-off point for PD of 8 ‘yes’ responses has been identified using validation data from the development of the SSQ; however, a lower cut-off point of 7 was used for this study as it was a large-scale population-based analysis which requires higher sensitivity.^24^ This cut-off point has also been used in the same setting previously.^17^ A new dichotomous variable was created in which participants who responded yes to 7 or more items from the 14-item tool were defined as having PD.

Smoking status, consumption of alcohol and use of drugs for pleasure were also assessed at baseline. Smoking was defined as responding ‘yes’ to the question ‘Do you smoke cigarettes?’. The use of drugs for pleasure was also defined as reporting taking any drugs for pleasure, regardless of whether participants took drugs by injection, ingestion or smoking. Consumption of alcohol was defined as visiting a bar regularly or having more than three beers per visit.

Participant demographic characteristics assessed at baseline included gender, age, marital status, level of education, employment status and study site type. To capture household socioeconomic status, a wealth index was determined from baseline data on sellable and non-sellable assets owned by the household.^29^ Sellable assets included radios, televisions, bicycles, motorbikes and cars, while non-sellable assets included water and electricity supply, toilet facilities, housing structure and floor type. To calculate the wealth index, each asset was given a score between 0 and 1. For example, a score of 0 was assigned for a natural floor (eg, sand), a score of 0.5 was assigned for a rudimentary type of floor (eg, planks) and a score of 1 was assigned for finished floors (eg, cement). The scores for each asset were summed and divided by the total number of assets to create the wealth index. This distribution was then split into four equally sized groups (least wealthy, less wealthy, more wealthy, most wealthy). Data from every individual were then matched to the wealth of their household for analysis.

Hypertension was assessed as a self-reported variable at follow-up in round 7. Participants responded ‘yes’ or ‘no’ to the question: ‘Have you ever been diagnosed with hypertension by a medical doctor or nurse?’.

### Data analysis

The proportions of participants reporting a range of sociodemographic characteristics were calculated, stratified by whether participants reported PD at baseline. PD prevalence at baseline and cumulative incidence of hypertension at follow-up were measured. χ^2^ tests for comparison were performed to describe the study population.

Logistic regression was used to estimate the association between PD and later development of hypertension. First a set of univariable models were developed, which explored the relationship between a series of potential risk factors for hypertension, including PD and hypertension. The univariate model that explored the relationship between PD and hypertension was identified as model A. Next, a set of multivariable models were developed that focused on the association between PD and hypertension, using a forward inclusion method. These included a model adjusted for age and gender (model B), a model adjusted for age, gender and the sociodemographic risk factors identified in the univariate analysis (model C), and a model adjusted for all the previous sociodemographic covariates, as well as alcohol consumption, smoking and drug use (model D). The final two models—with and without health risk behaviours—were then compared with assess the role of health risk behaviours in the relationship between PD and hypertension. Analyses were complete case analyses, with statistical significance assessed at p<0.05, and performed in STATA V.16.

### Patient and public involvement

The public provided input into the design of the Manicaland study through small meetings held with community members at the start of the study and through providing feedback at dissemination meetings that were held to share results from successive rounds of the study. Members of the public provided recommendations on the research questions, outcome measures, and recruitment and conduct of the study, which were incorporated into the study procedures. In addition, local guides (mainly community health workers) provided continuous feedback as the study progressed, and local team members also participated in formulating the detail of the study design.

The results of this study will be disseminated to local stakeholders and community members through feedback meetings, as part of a wider plan to share results from the Manicaland Study. Involvement of community members and local team members will be crucial to the development of the dissemination strategy.

## Results

### Participants’ characteristics

Two-thirds of the sample identified as female (68.6%). Most of the participants were under the age of 35 (65.1%), married (70.1%), unemployed (69.1%) and had completed secondary or higher education (62.8%). Around 16% of the participants were in the lowest wealth quartile (table 1) and almost half (49.7%) were from rural areas.

**Table 1:** Sociodemographic characteristics and prevalence of psychological distress (PD) at baseline among 742 adult participants in the Manicaland study who were included in the analysis of the association between PD and later development of hypertension

	Population distribution		Prevalence of PD	
	Proportion (%)	No of participants (N)	Proportion (%)	P value*
Gender				<0.001
Male	31.4	233	4.3	
Female	68.6	509	13.1	
Age group				0.021
<35 years	65.1	483	8.5	
≥35 years	34.9	259	13.9	
Marital status				0.006
Never married	10.6	79	3.8	
Married	70.1	520	9.6	
Separated, divorced or widowed	19.2	143	16.8	
Site type				0.600
Periurban	25.3	188	12.2	
Agricultural estates	25.0	185	10.3	
Rural	49.7	369	9.5	
Level of education				0.002
Primary and below	37.2	276	14.8	
Secondary and above	62.8	466	7.7	
Employment status				0.210
Unemployed	69.1	513	11.3	
Employed	30.9	229	8.3	
Wealth index				0.150
Least wealthy	16.4	122	12.3	
Less wealthy	41.6	308	11.4	
More wealthy	30.3	225	10.7	
Most wealthy	11.7	87	3.4	
Smoking				0.190
No	90.7	673	10.8	
Yes	9.3	69	5.8	
Alcohol consumption				0.051
No	83.0	616	11.4	
Yes	17.0	126	5.5	
Use of drugs for pleasure				0.650
No	92.2	684	10.5	
Yes	7.8	58	8.6	

*P value is derived from a χ^2^ test comparing participants with PD to participants without PD.

Of the 742 sample participants, 10.4% (77/742) had PD at baseline. This was similar to the prevalence of PD in all survey participants in 2012–2013 (8.9%, 549/6,187), prior to the selection of the analytic sample for this study.

### Cumulative incidence of hypertension

At follow-up (after 6 years on average), 17.5% of the 742 sample participants reported that they had been told by a doctor or nurse that they had hypertension. New reports of hypertension were more common among the 77 participants with PD at baseline (33.8%) than among the 665 participants without PD at baseline (15.6%) at baseline (χ^2^ test p<0.001).

### Risk factors for hypertension

The univariate analyses exploring the relationship between each sociodemographic covariate and hypertension highlighted several potential risk factors for hypertension (table 2). Females had 6.15 (95% CI 3.24 to 11.67) times greater odds of reporting a new hypertension diagnosis by doctor or nurse at follow-up, compared with males. Participants aged 35 years and over had 3.29 (95% CI 2.23 to 4.87) times higher odds of being diagnosed with hypertension compared with those younger than 35 years. Not having ever been married was associated with lower odds of being diagnosed (OR 0.2, 95% CI 0.06 to 0.63) compared with being currently married, whereas, divorced, separated or widowed people had 93% higher odds of being diagnosed with hypertension (OR 1.93, 95% CI 1.25 to 2.98). With every quartile increase in the wealth index, the odds of hypertension also increased, although there was not a large difference between the highest wealth index quartiles.

**Table 2:** Univariate analysis of the odds of being diagnosed with hypertension among adult participants in the Manicaland study between 2012–2013 and 2019

	No of participants (N)	Proportion who reported a hypertension diagnosis (%)	Crude OR (95% CI)	P value
Psychological distress				
No (ref)	665	15.64	1	
Yes	77	33.77	2.75 (1.64 to 4.60)	<0.001
Gender				
Male (ref)	233	4.72	1	
Female	509	23.38	6.15 (3.24 to 11.67)	<0.001
Age group				
< 35 years (ref)	483	11.18	1	
≥ 35 years	259	29.34	3.29 (2.23 to 4.87)	<0.001
Marital status				
Married (ref)	520	16.73	1	
Never married	79	3.80	0.2 (0.06 to 0.63)	0.007
Separated/widowed /divorced	143	27.97	1.93 (1.25 to 2.98)	0.003
Site type				
Peri-urban (ref)	188	15.96	1	
Agricultural estates	185	15.14	0.93 (0.53 to 1.64)	0.827
Rural	369	19.51	1.27 (0.79 to 2.03)	0.306
Level of education				
Primary and below (ref)	276	26.45	1	
Secondary and above	466	12.23	0.38 (0.26 to 0.56)	<0.001
Occupation				
Unemployed (ref)	513	18.91	1	
Employed	229	14.41	0.72 (0.46 to 1.10)	0.138
Wealth index				
Least wealthy (ref)	122	10.66	1	
Less wealthy	308	16.56	1.66 (0.87 to 3.18)	0.124
More Wealthy	225	20.89	2.21 (1.15 to 4.28)	0.018
Most wealthy	87	21.84	2.34 (1.09 to 5.05)	0.03
Health risk behaviours				
Smoking				
No (ref)	673	18.57	1	
Yes	69	7.25	0.34 (0.13 to 0.86)	0.024
Alcohol consumption				
No (ref)	616	18.99	1	
Yes	126	10.32	0.49 (0.26 to 0.90)	0.022
Drug use for pleasure				
No (ref)	684	18.42	1	
Yes	58	6.90	0.32 (0.11 to 0.92)	0.035

PD at baseline was strongly associated with being diagnosed with hypertension in the following years (OR 2.75, 95% CI 1.64 to 4.60). However, an individuals’ odds of being diagnosed with hypertension at follow-up were lower among participants who reported health risk behaviours including smoking (OR 0.34, 95% CI 0.13 to 0.86), alcohol consumption (OR 0.49, 95% CI 0.19 to 0.28) and use of drugs for pleasure (OR 0.32, 95% CI 0.11 to 0.92) at baseline.

### Association between PD and later development of hypertension

After adjusting for significant sociodemographic covariates from univariate analyses and health risk behaviours (model D), those with PD had 2.04 times greater odds of developing hypertension in the following years compared with those without PD (table 3).

**Table 3:** Comparison of unadjusted and adjusted multivariable models for the association between psychological distress and later development of hypertension

	Model	OR (95% CI)	P value	Akaike information criterion	Bayesian information criterion
Unadjusted	Model A	2.75 (1.64 to 4.60)	<0.001	679.22	688.43
Adjusted for age and gender	Model B	1.93 (1.12 to 3.37)	0.019	607.65	626.09
Adjusted for age, gender, marital status, education and wealth index	Model C	2.01 (1.14 to 3.54)	0.015	606.27	652.37
Adjusted for all above demographic covariates+alcohol+smoking+drugs	Model D	2.04 (1.16 to 3.59)	0.013	610.56	670.48

### Role of health risk behaviours in the relationship between PD and later development of hypertension

The final multivariable model (model D) was compared with the model without health risk behaviours (model C), to assess the role of health risk behaviours in the relationship between PD and hypertension (table 4). When added to the model, smoking, alcohol consumption and drug use for pleasure were not associated with self-reported hypertension diagnosis and the association between PD and self-reported hypertension diagnosis was largely unchanged.

**Table 4:** Comparison of two multivariable models (model C and model D) to assess the role of health risk behaviours in the relationship between psychological distress and hypertension

	Multivariable model C (without health risk behaviours)		Multivariable model D (with health risk behaviours)	
	Adjusted OR (95% CI)	P value	Adjusted OR (95% CI)	P value
Psychological distress				
No (ref)	1		1	
Yes	2.01 (1.15 to 3.54)	0.015	2.04 (1.16 to 3.59)	0.013
Gender				
Male (ref)	1		1	
Female	5.57 (2.82 to 11.02)	<0.001	6.89 (2.71 to 17.53)	<0.001
Age group				
<35 years (ref)	1		1	
≥35 years	2.72 (1.66 to 4.45)	<0.001	2.67 (1.63 to 4.42)	<0.001
Marital status				
Married (ref)	1		1	
Never married	0.43 (0.13 to 1.47)	0.179	0.45 (0.13 to 1.53)	0.202
Separated/widowed/divorced	1.06 (0.66 to 1.72)	0.811	1.06 (0.66 to 1.73)	0.797
Level of education				
Primary and below (ref)	1		1	
Secondary and above	0.75 (0.46 to 1.22)	0.254	0.76 (0.46 to 1.23)	0.266
Wealth index				
Least wealthy (ref)	1		1	
Less wealthy	1.31 (0.66 to 2.61)	0.439	1.29 (0.65 to 2.57)	0.468
More wealthy	2.13 (1.06 to 4.29)	0.034	2.10 (1.04 to 4.24)	0.038
Most wealthy	2.91 (1.26 to 6.72)	0.012	2.88 (1.24 to 6.67)	0.014
Health risk behaviours				
Smoking				
No (ref)	_	_	1	
Yes	_	_	1.41 (0.40 to 4.98)	0.591
Alcohol consumption				
No (ref)	_	_	1	
Yes	_	_	1.49 (0.61 to 3.66)	0.381
Drug use for pleasure				
No (ref)	_	_	1	
Yes	_	_	0.49 (0.14 to 1.75)	0.271

## Discussion

To the best of our knowledge, this is the first longitudinal study in sub-Saharan Africa, outside of South Africa, that both examines the relationship between PD and later development of hypertension and assesses the role of health risk behaviours in this relationship. Within our general population cohort in eastern Zimbabwe, the cumulative incidence of hypertension after 6 years was 17.5%, and the odds of being diagnosed with hypertension almost doubled for those with PD. We also found evidence that female gender, older age and higher wealth were significant risk factors for hypertension. However, the health risk behaviours that we studied did not appear to have a substantial role in the relationship between PD and hypertension.

The prevalence of PD in our analytical sample (10.4%) was similar to that in the entire cohort at baseline (8.9%), although this was not confirmed with a statistical test. However, both these estimates are lower than in a cross-sectional population study in South Africa where PD prevalence was 17.3%, measured using the Kessler Psychological Distress (K10) scale.^10^ Our estimate of the cumulative incidence of self-reported hypertension over a 6-year period in east Zimbabwe (17.5%) was also lower than the cumulative incidence after 5 years of follow-up in another study in South Africa (23%–24%).^11 30^ Differences in measurement tool, misreporting, lost to follow-up and recall bias due to self-reporting and ineffective health-seeking behaviours could be reasons for these dissimilarities.^13 31^

Among sociodemographic factors, female gender, older age and higher wealth were found to be risk factors for self-reported hypertension diagnosis. This is consistent with much previous research in settings including Zimbabwe, and several explanations have been proposed for how each risk factor affects hypertension.^18 32–35^ For example, in the Zimbabwean context, women are more likely to be overweight and physically inactive than men, and tend to have more active health-seeking behaviour, increasing the probability of reporting hypertension.^13 36–40^ Similarly, with increasing age, physiological changes and a more sedentary lifestyle mean that an individual is at a higher risk for morbidities.^41–43^ Moreover, experiencing menopause decreases the protective effect of oestrogen which in turn contributes to a higher risk for hypertension among older age women.^44 45^ Finally, wealth-related inequalities in proximal risk factors such as obesity, as well as differences in awareness of hypertension and access to care, are a probable explanation for the association between wealth and hypertension in Zimbabwe.^46–50^ The identification of gender, age and wealth as being associated with hypertension in this study emphasises how hypertension control programmes should continue to consider more traditional sociodemographic risk factors alongside PD.

In addition to sociodemographic risk factors, this study looked at three health risk behaviours. We found that the OR for the relationship between PD and hypertension did not differ substantially between the models with and without the three health risk behaviours. This is an indication that these three health risk factors may not be a key part of the causal pathway from PD to hypertension. Other health risk factors, such as diet and exercise levels, may have a more central role, especially given the comparatively low prevalence of smoking, alcohol consumption and drug use.^38 51^

This study has several strengths. These include an analytical sample drawn from a prospective general population cohort which allowed for assessment of the temporal association between PD (measured at baseline) and hypertension diagnosis (measured at follow-up). This study design limits the role of reverse causation as participants were selected before the disease outcome. In addition, the long follow-up duration of an average of 6 years provided more person years of observation to measure the effect of PD on hypertension.

However, there are limitations to this study too. Having a longer follow-up time also meant greater out-migration of participants from the study areas. Additionally, both the health risk behaviours and disease outcome were determined using self-reports, which means there might be under-reporting or over-reporting of the disease; this is a common problem with measuring self-reported health outcomes and behaviours in population studies.^52^ Using self-reports also introduces the potential for measurement bias as those with PD may be more likely to get themselves tested for hypertension compared with those without PD. Furthermore, although the sample was collected randomly from households there was lack of representation of urban sites, therefore, these results are more generalisable to rural populations. Finally, although the study analysed a range of predictor variables, there are some potential confounders which were not in the scope of this analysis that might have resulted in residual confounding.

We found a positive association between PD and development of hypertension. This result is consistent with findings from the existing South African studies, which also reported a positive association.^10 53^ Our study shows that PD may have an important role in development of hypertension, which should be investigated further to inform service provision to reduce morbidity and premature mortality. It is also clear that PD affects individual well-being, and may impact physical health, so should be considered when planning health service delivery. It is essential to include psychological health screening in primary healthcare and to sensitise healthcare professionals on the role of psychological health in addressing physical health symptoms. Several activities need to be scaled up for better mental health service delivery in Zimbabwe. These include training of healthcare professionals in mental health, increasing availability of psychiatric drugs, addressing the availability of the human resource, improving consultation time and quality, and increasing reliability of referral systems.^54^ There are multiple drivers of mental health in sub-Saharan Africa and to tackle the challenge, it is necessary to have a multipronged approach and involve all stakeholders in decision-making.

## Conclusion

PD is prevalent in many sub-Saharan African settings so understanding its effects on population health is vital. This study contributes much needed evidence on the temporal association between PD and later reports of hypertension in a sub-Saharan African setting. Within our cohort, individuals with PD had twice the risk of developing hypertension and female gender, older age and higher wealth were significant risk factors for hypertension. However, the health risk behaviours that we studied did not appear to have a substantial role in the relationship between PD and hypertension in the study population. Including mental health at the heart of hypertension prevention and control strategies will be key to reducing the burden of both diseases.

## Data Availability

Data are available on reasonable request. The data are deidentified participant data and can be obtained by sending a data request form (available from the Manicaland study website: http://www.manicalandhivproject.org/data.html) to s.gregson@imperial.ac.uk.
